# Avoidance-motivational intensity modulated the effect of negative emotion on working memory

**DOI:** 10.1098/rsos.221128

**Published:** 2023-06-07

**Authors:** Tianya Hou, Yawei Xie, Jianguo Zhang, Zhuoer Sun, Qianlan Yin, Ziqiang Li, Wenpeng Cai, Wei Dong, Guanghui Deng, Xiaofei Mao

**Affiliations:** Faculty of Psychology, Naval Medical University, Shanghai 200433, People's Republic of China

**Keywords:** avoidance-motivational intensity, working memory, maintenance, manipulation, negative effect

## Abstract

Although many studies have explored the association between negative emotion and working memory, the findings remain controversial. The present study investigated the role of avoidance-motivational intensity in modulating the effect of negative emotion on different processes (maintenance versus manipulation) of verbal and spatial working memory. Two experiments employed the modified delayed match-to-sample paradigms to separate the two processes of verbal and spatial working memory under different emotional states, respectively. In Experiment 1, participants were asked to perform the delayed match-to-sample task with or without reordering the characters (manipulation process of verbal working memory). In Experiment 2, mental rotation was used as the manipulation process of spatial working memory. The results showed that negative emotion only affected the manipulation process, but not the maintenance process. Relative to neutral and low avoidance-motivated negative conditions, the manipulation processes of both types of working memory were impaired under the high avoidance-motivated negative condition. No significant difference was observed between low avoidance-motivated negative condition and neutral condition. Our results are discussed in relation to efficiency processing theory and motivational dimensional model of affect. We conclude that negative emotional states with high avoidance-motivational intensity impair the manipulation process of verbal and spatial working memory.

## Public significance statement

The present study investigated the role of feeling of avoidance in modulating the effect of negative emotion on the different processes (maintenance versus manipulation) of verbal and spatial working memory. Two processes of verbal and spatial working memory were separated in Experiment 1 and Experiment 2, respectively. We used the paradigms to separate the two processes of verbal and spatial working memory. For both types of working memory, emotion was shown to only affect the manipulation process, but not the maintenance process of working memory. Only negative effective states with high avoidance-motivational intensity had disruptive effects on the manipulation process of both types of working memory, while negative states with low avoidance-motivational intensity had null effects. This suggests when you feel a negative emotion in daily life, it does not necessarily lead to a worse cognitive performance. Only when you experience a negative emotion and have a strong feeling of avoidance will your advanced cognitive activity be disrupted.

## 1. Introduction

Emotion and working memory play important roles in all aspects of daily life. There is increasing research interest in investigating the association between effective states and working memory. Working memory, as a vital component of advanced cognitive functions, consists of two distinct processes: maintenance and manipulation [1]. The former refers to the process that keeps information (e.g. storing and matching information) in an active state, while the latter involves the operation of information representations (e.g. mental rotation and reordering task) in mind over a short period of time [2,3]. Among various models of working memory, one commonly accepted model is Baddeley's multi-component model [1,4], which proposed that working memory consisted of four components: phonological loop, which maintained verbal information; the visuospatial sketchpad, which stored non-verbal information; episodic buffer, which could integrate information from various coding systems; and central executive, which was the core of working memory and was responsible for the executive functions. Thus, verbal and non-verbal information is processed in different systems.

Working memory has been widely investigated with well-established paradigms such as *N*-back [5,6], delayed match-to-sample task [7,8] and working memory span task [9,10]. Each paradigm focuses on the measurement of a certain aspect of working memory. For example, *N*-back is commonly employed to measure the updating ability of working memory. Delayed match-to-sample task is used to investigate the encoding, maintenance and retrieval processes of working memory. By contrast, the paradigms exploring the maintenance and manipulation processes of working memory have been less investigated. D'Esposito *et al*. [2] firstly put forward a modified delayed-response task including a maintenance process and a manipulation process (reordering) of verbal working memory. In the study conducted by Postle *et al*. [11], participants were asked to encode the location of highlighted squares in the order presented; then they were required to either keep spatial–temporal information in mind (maintenance condition) or reorder the spatial–temporal information (manipulation condition) according to the instruction. Glahn *et al*. [12] combined the spatial delayed-response task (maintenance) with a mental rotation task (manipulation) to dissociate the two processes of spatial working memory. Veltman *et al*. [13] used delayed match-to-sample task as a maintenance condition and *N*-back task as a manipulation condition to explore the two processes. In addition, previous literature also used *N*-back task to indicate the maintenance and manipulation through several contrasts [14–16]. Specifically, 1-back versus 0-back contrast revealed the maintenance process, while 2-back versus 1-back condition reflected the manipulation process. Collectively, there are some paradigms with respect to the maintenance and manipulation processes. However, no paradigm has been ‘universally’ used and there might be additional paradigms in this area. Reordering, updating and mental rotation are commonly used to measure the manipulation.

According to the task relevance of emotion, the research investigating the impact of emotion on working memory could be categorized into two types: incidental effect and integral effect [17–19]. The former explores the working memory performance under different emotional states and the stimuli in the working memory task are irrelevant to the effective states [17]. By contrast, the latter investigated how the emotional stimuli are processed in working memory and emotional information is a part of stimuli [17]. It is of great importance to differentiate between the incidental effect and integral effect since the task relevance of emotion could influence the effect of emotion on cognition [19–21]. In the present study, we focused on the incidental effect.

Over the past three decades, research on the effect of negative emotional states on working memory performance has become a hot topic. Eysenck & Calvo [22] found the mediating role of working memory in the effect of anxiety on cognitive performance and put forward processing efficiency theory. The theory proposed that anxiety mainly affected the central executive component of working memory, which started a chain reaction on the process of verbal and spatial information. Thus, both verbal and spatial working memory tasks might be impaired by anxiety. Many subsequent studies have provided evidence for the theory [23,24]. Lavric *et al*. [25] explored the impact of shock-induced anxiety on spatial and verbal working memory. There were two kinds of blocks: threat to shock and safe. In the threat to shock block, only one shock was administrated. The results presented that before the shock, only spatial working memory was impaired under the threat condition in comparison to safety condition. After the shock, both types of working memory were disrupted. A recent study conducted by Vytal *et al*. [26] showed anxiety disrupted both types of working memory. The anxiety-related impairment in verbal working memory was affected by cognitive load, while the impairment in spatial tasks was consistent across load. These suggested induced negative emotions disrupted both spatial and verbal working memory, and the deficits were greater in spatial tasks than verbal tasks. However, there are some studies showing contradictory results. Ikeda *et al*. [27] showed test-induced anxiety selectively disrupted verbal working memory performance but not spatial task, while some other studies found only spatial working memory was affected by anxiety [28,29]. The results from clinical studies involving patients with psychiatric disorders also contributed to the understanding of emotion–cognition link [28]. Findings from recent literature showed only spatial working memory was impaired in patients with bipolar II and unipolar depression [30]. From the previous literature, it can be concluded that researchers have not yet reached a consensus regarding the effect of negative emotion on working memory. This might be attributed to the fact that most studies focused on the impacts of emotional valence and arousal on working memory [20]. The inconsistency has suggested that only valence and arousal cannot fully explain the effects.

In 2010, the motivational dimensional model of effect put forward by Gable and Harmon-Jones suggested apart from valence and arousal, the motivational dimension was another dimension of emotion. Motivational intensity modulated the effect of emotion on cognitive performance [31]. Emotional state with low-motivational intensity could lead to cognitive broadening, while emotional state with high-motivational intensity could result in a narrowing of cognitive scope, which has proved adaptive. Attentional narrowing in negative states with high-motivational intensity could help individuals to evaluate and avoid disadvantageous situations. Attentional broadening in negative states with low-motivational intensity could help individuals to disengage from a blocked goal, find new solutions and promote resource conservation. Although several studies were conducted to examine the effect of motivational dimension on attentional scope, conflict resolution, visual word search and memory [32–35], only a handful of studies have explored the impact of motivational dimension of emotion on working memory. Gary [36] reported that approach state improved verbal working memory and impaired spatial working memory, while withdrawal state enhanced the spatial tasks and disrupted verbal tasks. However, another study suggested both approach and withdrawal states enhanced spatial working memory performance [37]. Recent research conducted by Yüvrük *et al*. [21] compared the effects of the motivational dimension and valence dimension on working memory under the control of arousal dimension and reported motivational dimension was more effective in explaining the effect of emotion on working memory speed. In sum, literature regarding the effects of emotion on working memory from the aspect of motivational dimension is limited and lacks proper control on irrelevant emotion dimensions.

To the best of our knowledge, only few studies have examined the effect of negative emotion on the maintenance and manipulation processes of working memory. A functional magnetic resonance imaging study conducted by Basten *et al*. [38] used a modified delayed-response task to dissociate working memory manipulation process from maintenance and explored the difference in neural efficiency of the maintenance and manipulation of working memory between low-anxious and high-anxious participants. The findings showed high-anxious participants presented decreased neutral efficiency than low-anxious participants only in the manipulation process, suggesting trait anxiety only influenced the manipulation of working memory. A recent study observed reduced activity of dorsolateral prefrontal cortex, which has been associated with manipulation process, in the threat condition during sort tasks [39], indicating threat disrupted the manipulation process of working memory.

## 2. Experimental aim

Given the contradictory findings in previous literature regarding the effect of negative emotional states on working memory performance, it is vital to clarify the role of avoidance-motivational intensity in the association. Hence, the present study aimed to answer three questions: (i) How do negative emotional states influence the maintenance and manipulation processes of working memory? (ii) How does avoidance-motivational intensity modulate the impact of negatively valenced effective state on working memory? (iii) Is the modulating effect of avoidance-motivational intensity the same for verbal and spatial working memory tasks?

Two experiments were carried out during which subjects were induced with high avoidance-motivated negative state, low avoidance-motivated negative state and neutral effective state via effective pictures and later completed verbal (Experiment 1) and spatial (Experiment 2) working memory tasks. The following hypotheses were made according to the previous literature and the motivational dimensional model of effect: (i) negative effective states only affected the manipulation process; (ii) compared with neutral condition, high avoidance-motivated negative state disrupted the manipulation of verbal and spatial working memory, while low avoidance-motivated negative state enhanced the manipulation of verbal and spatial working memory.

All studies were approved by the ethics committees of the Second Military Medical University and all participants gave informed consent. This study was not preregistered. The datasets used and/or analysed and study materials used during the current study are provided in the electronic supplementary material.

## 3. Piloting: selection of pictorial materials

### 3.1. Participants

The sample size was calculated using G*Power 3.1.9.4. The minimum sample size for this within-subject repeated-measures design was computed to be 28. Forty-nine right-handed subjects (23 males and 26 females) with a mean age of 21.47 (standard deviation (s.d.) = 2.68) participated in the study. All participants were required to rate three different types of emotional pictures: low avoidance-motivated negative pictures, high avoidance-motivated negative pictures and neutral pictures.

### 3.2. Materials and procedure

Forty-five emotional pictures were selected from the Chinese Affective Picture System (CAPS) in 2021. All pictures were processed with Photoshop software with a picture size of 433 × 315 pixels. The pictures were selected based on the previous literature [34]. Pictures of sad scenes could elicit a low avoidance-motivated negative state, while pictures of fearful scenes and faces were used to induce a high avoidance-motivated negative state. Moreover, household object pictures in life, such as cups, plates and pens, were used to induce neutral effective states.

The block-designed paradigm was programmed by E-prime 2.0 and shown by LENOVO computer screens. There were three blocks and only one dimension was rated in each block. Participants were asked to assess their subjective feelings to emotional pictures on the three dimensions on a nine-point Likert scale using the modified self-assessment Manikin (SAM) effective rating system: valence (1 = very unpleasant, 9 = very pleasant), arousal (1 = very calm, 9 = very excited) and motivational intensity (1 = strongest feeling of avoidance, 9 = strongest feeling of approach).

### 3.3. Results

The means and s.d. of valence, arousal and avoidance-motivational intensity for three types of emotional pictures are presented in table 1.

**Table 1.** Means and s.d. of emotional picture ratings.

	valence	arousal	avoidance-motivational intensity
neutral	5.116 ± 0.398	2.840 ± 1.409	5.161 ± 0.340
sadness	3.052 ± 0.974	5.628 ± 1.552	3.207 ± 0.856
fear	3.009 ± 1.088	5.696 ± 1.651	2.390 ± 0.766

A one-way repeated-measure ANOVA with picture type as a within-subject factor was performed for each dimension. Least significant difference (LSD) *post hoc* test was conducted when appropriate. On the valence rating, a significant main effect for picture type was reported (*F*_1.30,62.54_ = 142.33, *p* < 0.01, $\eta_{p}^{2}=0.75$). *Post hoc* comparisons showed that valence ratings were significantly lower for the sad pictures and fear pictures relative to neutral pictures (*p* < 0.01), indicating both sad and fear pictures were negatively valenced. There was no difference between sad and fear pictures (*p* = 0.57). Similarly, there was a significant effect for picture type on the arousal rating (*F*_1.36,65.35_ = 158.32, *p* < 0.01, $\eta_{p}^{2}=0.77$). Both sad and fear pictures had significantly higher arousal ratings than neutral pictures (*p* < 0.01). No difference between sad and fear pictures in the arousal ratings was found (*p* = 0.51). On the avoidance-motivational intensity ratings, the main effect of picture type was significant (*F*_2,96_ = 251.53, *p* < 0.01, $\eta_{p}^{2}=0.84$). Sad and fear pictures had lower rating scores in avoidance-motivational intensity than neutral pictures (*p* < 0.01), and fear pictures presented lower scores than sad pictures (*p* < 0.01), suggesting sad and fear pictures could induce avoidance-motivational states and the avoidance-motivational intensity was higher for fear pictures than sad pictures. In line with the previous literature, sad and fear pictures to evoke low versus high avoidance-motivated negative effective states were effective. In the following experiments, 45 pictures were used to induce different effective states. Specifically, 15 sad pictures, 15 fear pictures and 15 object pictures were used to induce low avoidance-motivated negative effective state, high avoidance-motivated negative effective state and neutral effective state, respectively.

## 4. Experiment 1

The modified delayed-response task as a well-validated and reliable measure of the maintenance and manipulation of verbal working memory was employed in Experiment 1. Despite previous literature having explored the effect of negative emotion on verbal working memory, the findings have not reached consensus. In addition, relatively little attention has been paid to the influence of negative effect on the different stages of verbal working memory. Furthermore, no previous literature has examined whether avoidance-motivational intensity modulated the impact of negative effect on verbal working memory performance. Therefore, the current study preliminarily examined these issues.

### 4.1. Method

#### 4.1.1. Participants

G*Power 3.1.9.4 was employed for the sample size calculation for a six-repeated-measure ANOVA with an alpha error of 0.05, a power of 80% and an effect size of 0.25. The estimation presented that 19 participants would be sufficient. Twenty-six students voluntarily participated in Experiment 1 (13 males and 13 females; mean age 22.3 years, s.d. = 3.07). All subjects reported normal or corrected-to-normal vision and were right-handed. The consent form was obtained from all participants.

#### 4.1.2. Task paradigm

The modified delayed-response task developed by D'Esposito *et al*. [2] was employed in the current study to measure the maintenance and manipulation of verbal working memory. A set of several items were shown simultaneously in a random order. Then an instruction cue was presented, followed by a probe. In the original version of the paradigm, the items presented were letters, and the instruction cues were ‘forward’ and ‘alphabetize’. The probe consisted of a letter and a number. The participants were required to determine whether the number would correctly represent the position of the letter in the sequence that was arranged according to the instruction cues.

Taking the cultural elements into consideration, the Chinese participants might be not familiar with the alphabet. Instead of the alphabet, the present study used heavenly stems as stimuli in the paradigm: ‘甲’, the first of the heavenly stems; ‘乙’, the second of the heavenly stems; ‘丙’, the third of the heavenly stems; ‘丁’, the fourth of the heavenly stems; ‘戊’, the fifth of the heavenly stems. Thus, similar to the alphabet, heavenly stems have a specific order and the ascending sequence of heavenly stems is ‘甲乙丙丁戊’. In the paradigm, three characters from heavenly stems firstly appeared on the screen (e.g. 丁甲丙). Then, one of the two kinds of instruction cues was shown during the delay phase. One instruction cue was ‘forward’, which indicated the maintenance of the sequence of the items during the delay period since participants only need to keep the stimuli in mind without any manipulation (e.g. 丁甲丙). The other was ‘ascending’, including the maintenance and manipulation of the sequence of the items during the delay period. Under this circumstance, participants were asked to not only keep the stimuli, but also rearrange the stimuli according to the ascending sequence of heavenly stems (e.g. 甲丙丁). In other words, apart from maintaining the items, participants needed to manipulate the sequence of the stimuli. In the probe stage, the participants were instructed to determine whether the number in the probe (e.g. 甲—2) would correctly represent the position of the heavenly stem in the sequence that was maintained or reordered based on the instruction cues.

#### 4.1.3. Procedure

Experiment 1 had a 2 (type of task: maintenance-only, maintenance-plus-manipulation) × 3 (type of emotion: low avoidance-motivated negative effective state, high avoidance-motivated negative effective state and neutral effective state) within-subject design.

Participants were seated in a comfortable chair in front of a computer screen at a distance of about 55 cm in a dimly lit, quiet room. The experimental paradigm programmed using E-prime 2.0 was block designed and presented by LENOVO computer screens. There were three blocks with their order counterbalanced across subjects and each block consisted of 60 trials (30 verbal maintenance trials and 30 verbal maintenance-plus-manipulation trials), resulting in a total of 180 trials. Within each block, only one type of emotional picture was presented.

**Figure 1.**
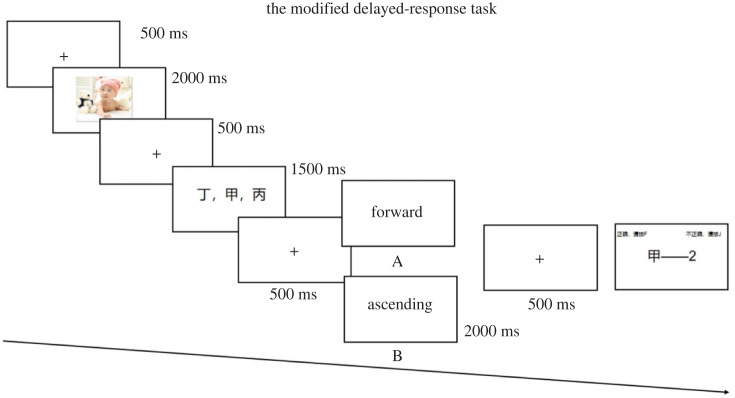
Experimental procedure of verbal working memory task. A represents a trial in the maintenance-only task; B represents a trial in maintenance-plus-manipulation task.

Figure 1 presents the experimental procedure of verbal working memory task. Each trial began with a black fixation cross (500 ms), followed by an effective picture from the pilot study (2000 ms) and a black fixation cross (500 ms). Then, three heavenly stems appeared (1500 ms) followed by a fixation cross (500 ms). Afterwards, instruction cue was displayed for 2000 ms, followed by a fixation cross (500 ms). A probe was presented, during which the participants responded with an ‘F’ (correct) or ‘J’ (wrong) button press. Before the formal experiment, a practice session with 10 trials was implemented.

### 4.2. Results

Within-subject 2 × 3 repeated-measures ANOVAs were conducted for the dependent variables: reaction times, accuracy and indexes established by signal detection theory (sensitivity and response bias)*.* Signal detection analysis was conducted to separate sensitivity from response bias, which could independently affect the accuracy [40]. This analysis was necessary when exploring which process was influenced by the effective states. Sensitivity (denoted as *d′*) represented the ability to identify the correct probe, while response bias measure (denoted as *c*) indicated the tendency towards a certain response. Sensitivity and response rate were calculated as follows:

$$\begin{array}{rl} & d^{\prime }=z(\mathrm{HR})-z(\mathrm{FA})\\ & c=-1/2[z(\mathrm{HR})+z(\mathrm{FA})]. \end{array}$$

HR refers to the hit rate (the proportion of the correct probe that the participants respond ‘correct’) and FA is the false-alarm rate (the proportion of the wrong probe that the participants respond ‘correct’). These proportions were corrected for extreme rates (maximum hit rates or null false-alarm rates) and then converted into z-scores.

Mauchly's test was employed to examine the assumption of sphericity. If the assumption was violated, Greenhouse–Geisser correction was performed. When significant interactions were found, simple effect analysis was conducted using LSD method.

#### 4.2.1. Working memory performance

A 2 × 3 repeated-measures ANOVA with task type and picture type as within-subject factors were conducted on reaction times data. Trials with incorrect response (6.35% on average) were excluded. The results showed significant main effects of task type (*F*_1,25_ = 15.76, *p* < 0.01, $\eta_{p}^{2}=0.39$) and picture type (*F*_2,50_ = 7.62, *p* < 0.01, $\eta_{p}^{2}=0.23$). The interaction between task type and picture type was significant (*F*_1.51,37.81_ = 5.50, *p* = 0.01, $\eta_{p}^{2}=0.18$).

**Figure 2.**
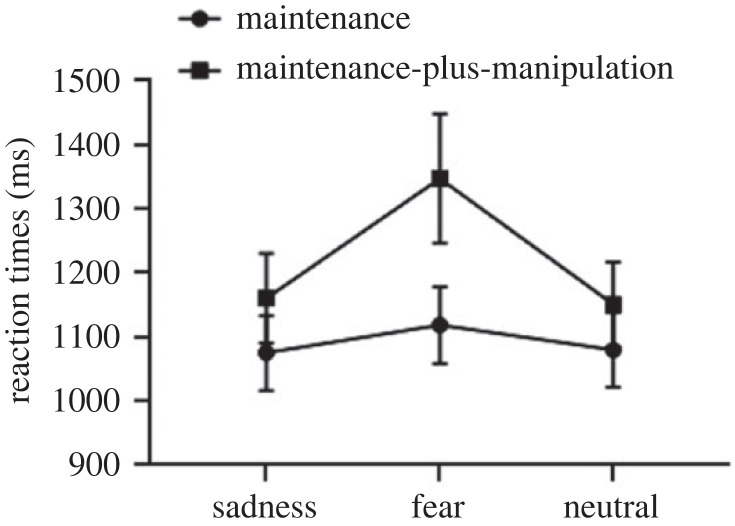
The reaction times on the maintenance-only and maintenance-plus-manipulation tasks of verbal working memory under induced-emotional states. Vertical bars indicate s.e. of the mean.

The interaction effect was further analysed using a simple effect analysis. We compared the reaction times under different emotional conditions during maintenance-only and maintenance-plus-manipulation processes. The results found that there was no significant effect for picture type during the maintenance-only task (*F*_1.40,34.95_ = 1.32, *p* = 0.27), indicating no difference between the sad (*M* = 1075.21, s.d. = 297.40), fear (*M* = 1118.66, s.d. = 305.78) and neutral (*M* = 1079.76, s.d. = 295.66) conditions. However, a significant difference between three conditions during maintenance-plus-manipulation processes was found (*F*_1.56,39.01_ = 8.48, *p* < 0.01, $\eta_{p}^{2}=0.25$). The reaction times under the fear condition (*M* = 1347.22, s.d. = 514.87) were longer than those under the sad (*M* = 1160.10, s.d. = 357.04) and neutral (*M* = 1150.76, s.d. = 337.50) conditions. There was no significant difference in reaction times between sad and neutral conditions. The results are presented in figure 2.

**Figure 3.**
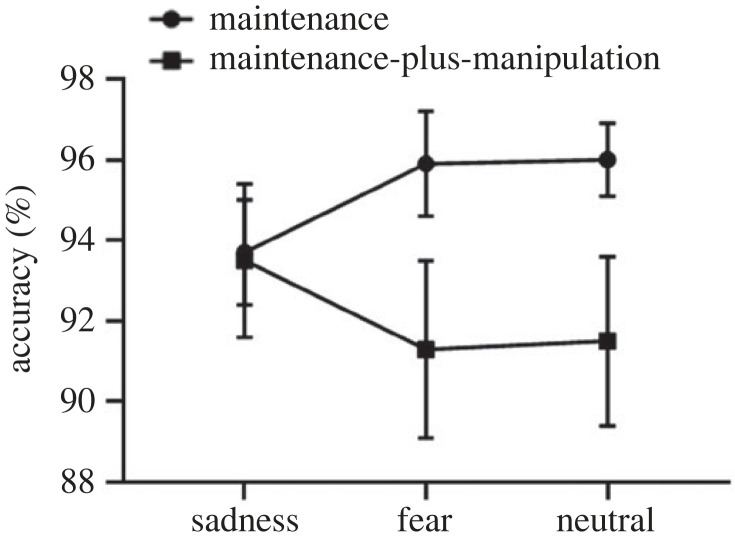
The accuracy on the maintenance-only and maintenance-plus-manipulation tasks of verbal working memory under induced-emotional states. Vertical bars indicate s.e. of the mean.

The 2 × 3 repeated-measures ANOVA on accuracy revealed a non-significant main effect of task type (*F*_1,25_ = 2.75, *p* = 0.11), a non-significant main effect of picture type (*F*_2,50_) = 0.03, *p* = 0.97) and a significant interaction effect between task type and picture type (*F*_2,50_ = 10.61, *p* < 0.01, $\eta_{p}^{2}=0.30$). Further simple effect analysis showed no significant difference in the accuracy of the maintenance-only process (*F*_2,50_ = 2.75, *p* = 0.07) between sad (*M* = 0.94, s.d. = 0.07), fear (*M* = 0.96, s.d. = 0.07) and neutral (*M* = 0.96, s.d. = 0.05) conditions. In addition, although the accuracy was lower in fear condition (*M* = 0.91, s.d. = 0.11) than that in sad (*M* = 0.93, s.d. = 0.10) and neutral (*M* = 0.92, s.d. = 0.11) conditions, the difference did not reach statistical significance during the maintenance-plus-manipulation process (*F*_2,50_ = 2.62, *p* = 0.08). The results are shown in figure 3.

**Figure 4.**
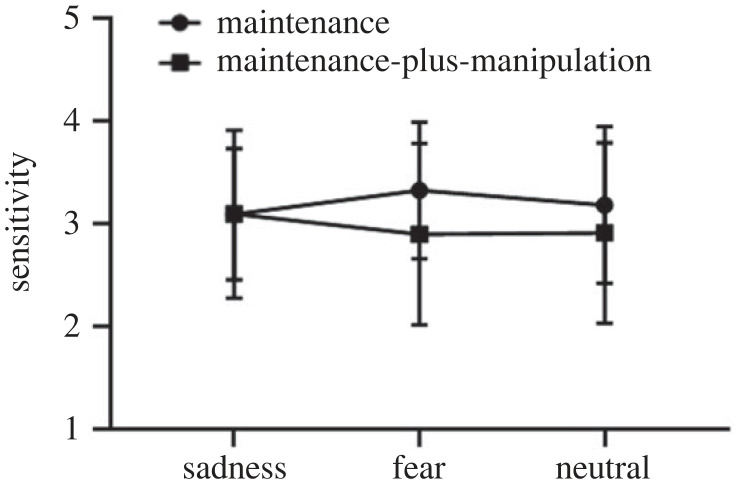
The sensitivity on the maintenance-only and maintenance-plus-manipulation tasks of verbal working memory under induced-emotional states. Vertical bars indicate s.e. of the mean.

The 2 × 3 repeated-measures ANOVA on sensitivity (figure 4) suggested a non-significant main effect of task type (*F*_1,25_ = 2.13, *p* = 0.16), a non-significant main effect of picture type (*F*_2,50_ = 0.22, *p* = 0.81) and a significant interaction effect between task type and picture type (*F*_2,50_ = 3.69, *p* = 0.03, $\eta_{p}^{2}=0.13$). Then, a simple effect analysis was conducted. During the maintenance-only task, no significant effect for picture type was found (*F*_1.63,40.72_ = 1.28, *p* = 0.28), suggesting no significant difference between the sad (*M* = 3.09, s.d. = 0.64), fear (*M* = 3.33, s.d. = 0.66) and neutral (*M* = 3.18, s.d. = 0.76) conditions. Likewise, there was no significant difference in the sensitivity during the maintenance-plus-manipulation process (*F*_2,50_ = 1.92, *p* = 0.16) between the sad (*M* = 3.09, s.d. = 0.82), fear (*M* = 2.90, s.d. = 0.88) and neutral (*M* = 2.91, s.d. = 0.88) conditions.

**Figure 5.**
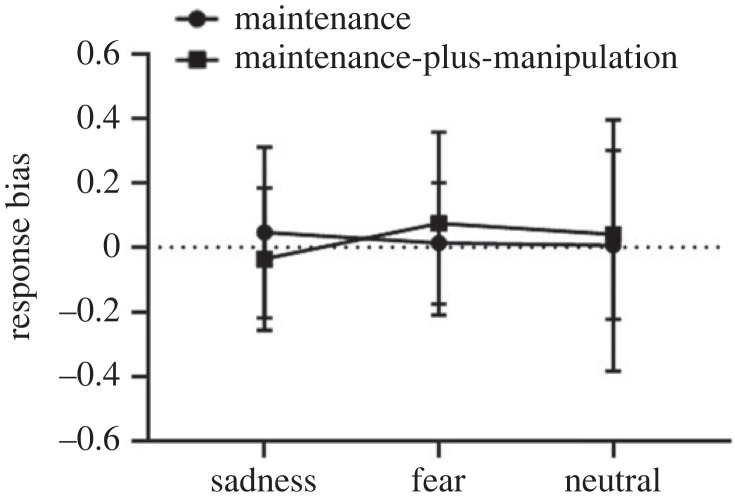
The response bias on the maintenance-only and maintenance-plus-manipulation tasks of verbal working memory under induced-emotional states. Vertical bars indicate s.e. of the mean.

For the response bias (figure 5), there were no significant main effects of task type (*F*_1,25_ = 0.01, *p* = 0.92) and picture type (*F*_2,50_ = 0.21, *p* = 0.81), and no significant interaction effect between task type and picture type (*F*_2,50_ = 1.29, *p* = 0.29).

### 4.3. Discussion

The reaction times during the maintenance-plus-manipulation process of verbal working memory were significantly longer than those during the maintenance-only process. The accuracy was lower for the maintenance-plus-manipulation task relative to the maintenance-only task, albeit the difference did not reach statistical significance. These results proved that maintenance-plus-manipulation task of verbal working memory was more difficult than maintenance-only task, and the Chinese version modified delayed-response task could reflect the characteristics of the maintenance and manipulation processes. The results of Experiment 1 presented that emotion did not affect the reaction times of maintenance-only trials and only influenced the reaction times of maintenance-plus-manipulation trials, suggesting only the manipulation process of verbal working memory was impacted by emotion. The fear condition had the longest reaction times during the maintenance-plus-manipulation task in comparison to the sad and neutral conditions, while no significant difference was observed in the reaction times between sad and neutral conditions. The findings indicated that, relative to the neutral and low avoidance-motivated negative conditions, high avoidance-motivated negative effective state had a detrimental effect on the manipulation of verbal working memory, whereas low avoidance-motivated negative effective state did not enhance or impair the performance. In the present study, the difference between sad and fear conditions could be attributed to the avoidance-motivational intensity since the valence and arousal dimensions of emotion have been controlled. Nevertheless, apart from motivation to avoid dimension, some researchers also used threat-relevance to interpret different influences of fear and sadness on cognitive performance [41]. There was no significant difference in accuracy, sensitivity and response bias between groups. In sum, negative emotion with high avoidance-motivational intensity impaired the manipulation of verbal working memory.

## 5. Experiment 2

The results of Experiment 1 elucidated the effect of negative emotion on the verbal working memory performance was modulated by the avoidance-motivational intensity. Nevertheless, it remains elusive whether the avoidance-motivational intensity could modulate the impact of negative effect on spatial working memory. In the second experiment, we used the modified delayed match-to-sample task paradigm to dissociate the maintenance and manipulation processes of spatial working memory. According to the previous literature [12], the manipulation of spatial working memory could be operationalized as mental rotation.

### 5.1. Method

#### 5.1.1. Participants

G*Power 3.1.9.4 was used to determine the sample size. Considering an α error equal to 0.05, a power equal to 80% and an effect size equal to 0.25, the sample size was estimated to be 19. Finally, 31 students participated in Experiment 2 (18 males and 13 females; mean age 22.6 years, s.d. = 3.74). All participants were right-handed and had normal or corrected-to-normal vision. All subjects signed the written informed consent.

#### 5.1.2. Task paradigm

Mental rotation has been used as the manipulation of spatial working memory task. In the paradigm, participants were shown three blue dots positioned in a circle pseudo-randomly and were asked to remember the location information of the three blue dots.

Then there were two kinds of instruction cues during the delay phase to indicate the maintenance-only task and maintenance-plus-manipulation task, respectively. During the delay period of the maintenance-only task, only a circle was presented as the instruction cue on the screen and participants only needed to keep the location information of the three blue dots in mind. During the delay period of the maintenance-plus-manipulation task, a circle with a mental rotation instruction appeared as the instruction cue and participants were asked to mentally rotate the dots 90° clockwise inside the circle. Under this circumstance, participants were required to not only maintain the location information, but also mentally rotate the information.

Then, a probe stimulus containing a blue dot in a circle appeared after the delay period. In the maintenance-only task, participants were required to indicate whether the blue dot in the probe stimulus was in the same position as one of the three dots presented previously. In the maintenance-plus-manipulation task, subjects were asked to indicate whether the blue dot in the probe stimulus was in the same position as one of the three rotated dots.

#### 5.1.3. Procedure

**Figure 6.**
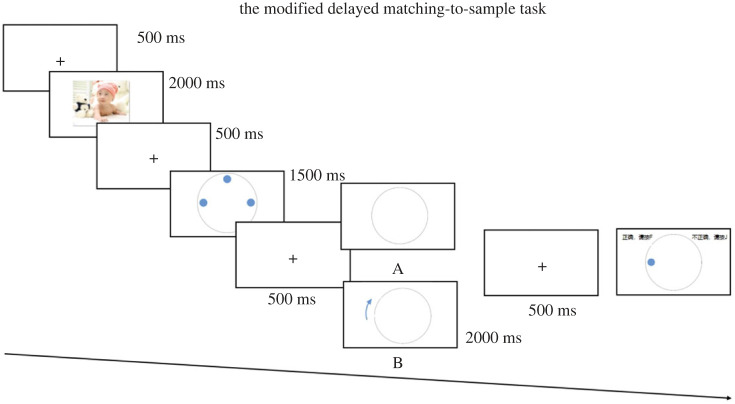
Experimental procedure of spatial working memory task. A represents a trial in the maintenance-only task; B represents a trial in maintenance-plus-manipulation task.

Experiment 2 has the same procedure as Experiment 1 with the exception of working memory task. The experimental procedure of spatial working memory task is presented in figure 6. Spatial working memory trials started with a black fixation cross shown for 500 ms, followed by an emotional picture from the pilot study presented for 2000 ms and a fixation cross for 500 ms. Then, a circle with three blue dots inside were shown for 1500 ms. The instruction cue preceded by a fixation cross (500 ms) appeared for 2000 ms, followed by a fixation cross (500 ms). Afterwards, a probe stimulus was presented and participants responded by pressing the ‘F’ key or ‘J’ key on the keyboard.

### 5.2. Results

As in Experiment 1, trials with incorrect responses (5.57% on average) were excluded from the analysis of reaction times. Reaction times were subjected to a 2 × 3 repeated-measures ANOVA, with picture type and task type as within-subject variables. The results showed a significant main effect of task type (*F*_1.56,46.77_ = 9.16, *p* = 0.01, $\eta_{p}^{2}=0.23$), a significant main effect of picture type (*F*_1,30_ = 28.64, *p* < 0.01, $\eta_{p}^{2}=0.49$) and a significant interaction effect (*F*_2,60_ = 11.58, *p* < 0.01, $\eta_{p}^{2}=0.28$). A simple effect analysis was conducted to further analyse the interaction effect of picture type and task type.

**Figure 7.**
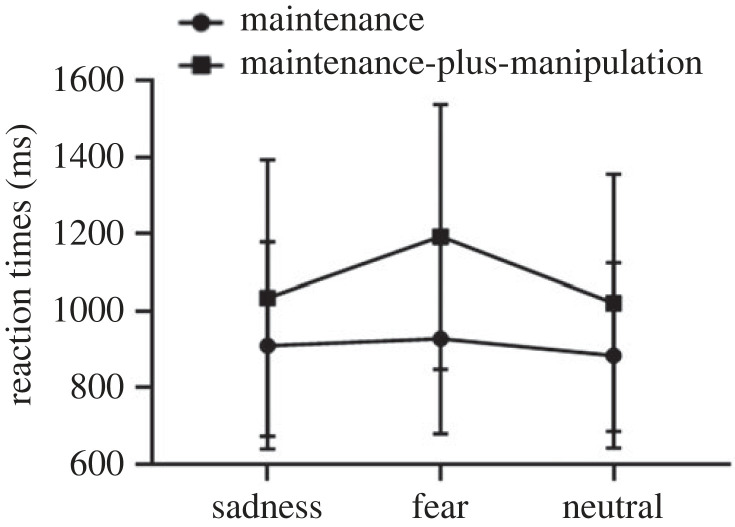
The reaction times on the maintenance-only and maintenance-plus-manipulation tasks of spatial working memory under induced-emotional states. Vertical bars indicate s.e. of the mean.

The reaction times under different emotional conditions during the maintenance-only and maintenance-plus-manipulation tasks were compared. The results revealed no significant difference in reaction times of the maintenance-only task (*F*_2,60_ = 1.55, *p* = 0.22, $\eta_{p}^{2}=0.05$) between the sad (*M* = 909.70, s.d. = 269.14), fear (*M* = 927.71, s.d. = 247.78) and neutral (*M* = 883.80, s.d. = 241.14) conditions. Nevertheless, a significant effect of picture type during the maintenance-plus-manipulation task was reported (*F*_1.48,44.38_ = 13.68, *p* < 0.01, $\eta_{p}^{2}=0.31$). Specifically, the reaction times in the fear mood induction group (*M* = 1192.50, s.d. = 344.35) were longer than those in the sad (*M* = 1033.42, s.d. = 359.42) and neutral (*M* = 1020.82, s.d. = 334.48) mood induction groups. No significant difference was observed between sad condition and neutral condition. The results are presented in figure 7.

**Figure 8.**
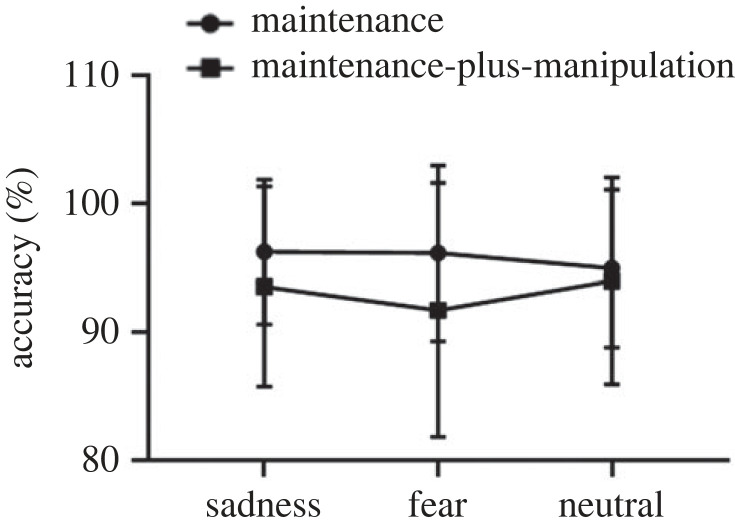
The accuracy on the maintenance-only and maintenance-plus-manipulation tasks of spatial working memory under induced-emotional states. Vertical bars indicate s.e. of the mean.

The repeated-measures ANOVA on the accuracy (figure 8) presented a non-significant main effect of task type (*F*_1,30_ = 2.83, *p* = 0.10), a non-significant main effect of picture type (*F*_2,60_ = 0.69, *p* = 0. 51) and a non-significant interaction effect (*F*_1.57,47.05_ = 2.66, *p* = 0.09).

**Figure 9.**
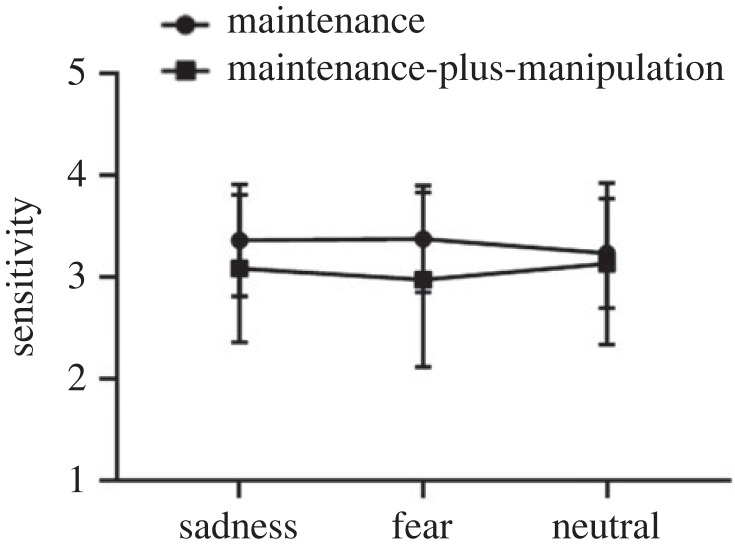
The sensitivity on the maintenance-only and maintenance-plus-manipulation tasks of spatial working memory under induced-emotional states. Vertical bars indicate s.e. of the mean.

For sensitivity (figure 9), the results of the repeated-measures ANOVA showed an insignificant main effect of task type (*F*_1,30_ = 3.61, *p* = 0.07), an insignificant main effect of picture type (*F*_2,60_ = 0.25, *p* = 0.78) and an insignificant interaction effect (*F*_1.58,47.43_ = 2.21, *p* = 0.13).

**Figure 10.**
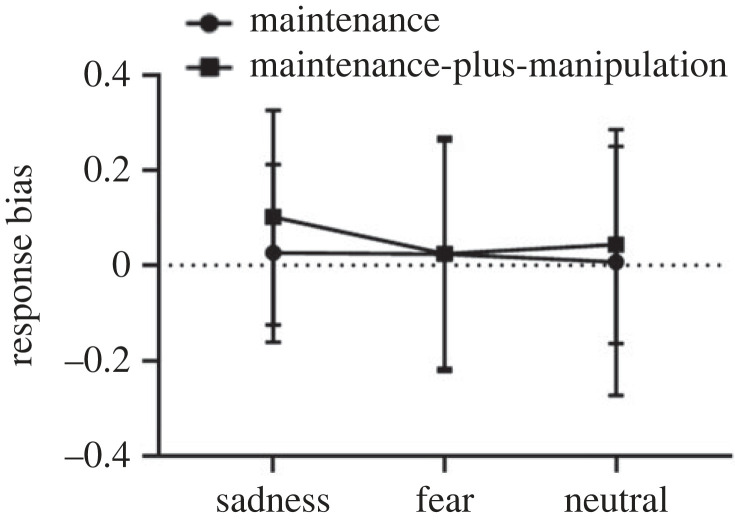
The response bias on the maintenance-only and maintenance-plus-manipulation tasks of spatial working memory under induced-emotional states. Vertical bars indicate s.e. of the mean.

The within-subject 2 × 3 repeated-measures ANOVA on the response bias (figure 10) revealed a non-significant main effect of task type (*F*_1,30_ = 0.78, *p* = 0.38), a non-significant main effect of picture type (*F*_2,60_ = 0.93, *p* = 0.40) and a non-significant interaction effect (*F*_2,60_ = 0.79, *p* = 0.46).

### 5.3. Discussion

For the spatial working memory task, shorter reaction times and higher accuracy are found in maintenance-only trials relative to maintenance-plus-manipulation trials. These illustrated the paradigms in our study meet the characteristics of the maintenance and manipulation of spatial working memory. In line with Experiment 1, an interaction effect of task type and picture type was found for reaction times. Emotional states only impacted the maintenance-plus-manipulation trials, suggesting effective states only influenced the manipulation of spatial working memory. There was no significant difference in the reaction times of the maintenance-plus-manipulation task between sad condition and neutral condition. The reaction times for fear condition were longer than those for sad and neutral conditions. Regarding accuracy, sensitivity and response bias, there were no main effects of picture type and task type, and interaction effect. Thus, among negative emotional states, only those with high avoidance-motivational intensity disrupted the manipulation of spatial working memory, while those with low avoidance-motivational intensity did not affect the manipulation process.

## 6. General discussion

Regarding the incidental effects of negative effective states on working memory performance, there have been contradictory findings in the extant literature. According to a recent model suggesting motivational intensity of emotion could modulate the emotion–cognition link, we conducted two experiments to explore the impact of negative emotional states varying in motivational intensity on the different stages of different types of working memory.

In both verbal and spatial tasks, we observed a prolonged reaction time in maintenance-plus-manipulation task in comparison to maintenance-only task. This is consistent with the previous literature [38,42], suggesting the differences between two tasks reflect manipulation process.

Consistent with our hypothesis and previous literature [38,39], only the manipulation process of working memory was affected by negative emotion with different avoidance-motivational intensity. Our results were divergent from the findings from other studies, which claimed the maintenance process was affected by emotion. Martin *et al*. reported the storage of working memory was affected by video-induced emotions. Running memory span was employed as working memory storage task. However, the task not only measured the maintenance process, but also the updating ability, which involved the manipulation process [43]. Thus, the disparity between our findings and the previous research might be attributed to the differences of the tasks, which contained different working memory processes. In addition, a recent study using delayed match-to-sample paradigm explored the effect of emotion on working memory maintenance and reported the detrimental effect of negative emotion on the maintenance. The use of strategy might account for the difference between the previous findings and the present study [44]. For instance, during the maintenance process, individuals might employ chunking strategies to retain information, which might result in the involvement of manipulation process in the maintenance task.

We found no significant difference in working memory performance between neutral state and negative emotional states with low avoidance-motivational intensity, which contradicted our hypothesis that low-motivational intensity facilitated the performance. As compared to neutral state and low avoidance-motivated negative effective state, high avoidance-motivated negative effective state disrupted the manipulation process of working memory. Negative and neutral emotions differed in all three dimensions of emotion, whereas high and low avoidance-motivated negative emotions only differed in the avoidance-motivated intensity. Thus, the results confirmed that high avoidance-motivational intensity impaired working memory performance. The findings could be explained as follows. According to processing efficiency theory, the process of negatively valenced emotions could occupy cognitive resources, and the limited resources were less available for the cognitive tasks. Thus, negative emotional states hindered the cognitive performance. Meanwhile, the motivational dimensional model of effect pointed out the low-motivational intensity facilitated cognitive performance, while high-motivational intensity reduced cognitive performance due to the relevance to survival. Valence and motivation are two independent dimensions of emotion. In the low avoidance-motivated negative condition, the detrimental effect of negative emotion and the facilitating effect of low-motivational intensity might cancel each other out, resulting in no statistically significant effect of negative emotion with low-motivational intensity. The harmful effects of negative emotion and high-motivational intensity combined and resulted in the impairment of working memory performance. Our result was consistent with recent literature on comparing the effects of fear and sadness on working memory performance. A recent study explored the filtering of task-irrelevant threatening faces (fearful) and task-irrelevant non-threatening faces (sad) when performing working memory tasks [45]. The results showed healthy adults could filter sad face distractors, but failed to filter fearful face distractors, indicating a threat-related filtering difficulty in the normal population. This suggested that task-irrelevant fearful stimuli would consume working memory resources, while task-irrelevant sad stimuli would not. However, the study compared the effects of fear and sadness on working memory performance from the perspective of threat-relevance. Our study goes beyond the previous literature by comparing fear and sadness from the perspective of motivational intensity with the control of valence and arousal.

In our study, both spatial and verbal working memory were impaired by negative effective states with high avoidance-motivational intensity, which is in accordance with processing efficiency theory [22]. While it would be tempting to conclude that spatial and verbal working memory were affected equally by emotional states, the current data alone are not sufficient to support this claim. The paradigms for exploring verbal and spatial working memory are not directly comparable in our study since they were not psychometrically matched. The two tasks used different materials and the accuracy of spatial working memory task was higher than that of verbal task in the present study. There is a possibility that negative effective states disrupt both types of working memory, and the impairment was greater in one specific type of working memory than the other type.

In the present study, apart from overall accuracy, signal detection metrics were reported: sensitivity and response bias, which could independently influence accuracy [40]. It is interesting to note in both tasks, the impairment effects of high avoidance-motivational intensity on accuracy, sensitivity and response bias were not observed. This is consistent with some existing literature, which has also reported the effect of emotion on the reaction times of working memory task, not on accuracy [21,39,46]. This could be explained by attentional control theory [23]. Under disadvantageous conditions, participants would sacrifice speed (reaction time) for accuracy.

Taken together, our results not only confirmed the processing efficiency theory, but also provided robust evidence for the motivational dimensional model of effect, which suggested motivational dimension was independent of valence.

## 7. Strengths and limitations

The key strength of the research is that we examined the role of avoidance-motivational intensity in modulating the association between emotion and working memory performance. Another strength of the study is that we separate the maintenance and manipulation components of both verbal and spatial working memory.

The main limitation of the current work is that the degree to which participants were emotionally evoked was not measured to evaluate the effectiveness of emotional induction in the present study. Nevertheless, it is worth noting that a block design in which only one type of effective pictures is included has been used in the present study and other previous literature as a robust way to induce emotions [28,44]. In addition, the current research explored the effects of task-irrelevant emotion on the processing of neutral stimuli in verbal and spatial working memory tasks. However, in real life, participants usually process emotional information under effective states. Thus, further studies could explore the incidental and integral effects of emotion on working memory concurrently. Finally, based on the motivational dimensional model of effect, the present study used fear and sadness to explore the effects of avoidance-motivated negative emotions on working memory and found that fear exerted a negative influence on the manipulation of working memory performance. However, research on the effects of other negative emotions with high avoidance-motivational intensity is still needed to further verify our result.

## 8. Conclusion

To conclude, the present research has revealed the role of avoidance-motivational intensity in modulating the effect of negative emotional states on the different stages of verbal and spatial working memory performance. For both verbal and spatial working memory, emotion was shown to only affect the manipulation process, but not the maintenance process of working memory. Only negative effective states with high avoidance-motivational intensity had disruptive effects on the manipulation process of both types of working memory, while low avoidance-motivational negative states had null effects.

## Data Availability

The datasets used and/or analysed during the current study are available from the corresponding author on reasonable request. In addition, the raw data are available on Figshare at https://doi.org/10.6084/m9.figshare.21127795 [47]. Electronic supplementary material is available online [48].
